# Fat-free mass prediction equations for bioelectric impedance analysis compared to dual energy X-ray absorptiometry in obese adolescents: a validation study

**DOI:** 10.1186/s12887-015-0476-7

**Published:** 2015-10-15

**Authors:** Geesje H. Hofsteenge, Mai JM Chinapaw, Peter JM Weijs

**Affiliations:** Department of Nutrition & Dietetics, Internal Medicine, VU University Medical Center, De Boelelaan 1117, 1081, HV Amsterdam, The Netherlands; EMGO Institute for Health and Care Research, VU University Medical Center, Amsterdam, The Netherlands; Department of Public and Occupational Health, VU University Medical Center, Amsterdam, The Netherlands; Department of Nutrition & Dietetics, Amsterdam University of Applied Sciences, Amsterdam, The Netherlands

**Keywords:** Body composition, Accuracy, Obesity

## Abstract

**Background:**

In clinical practice, patient friendly methods to assess body composition in obese adolescents are needed. Therefore, the bioelectrical impedance analysis (BIA) related fat-free mass (FFM) prediction equations (FFM-BIA) were evaluated in obese adolescents (age 11–18 years) compared to FFM measured by dual-energy x-ray absorptiometry (FFM-DXA) and a new population specific FFM-BIA equation is developed.

**Methods:**

After an overnight fast, the subjects attended the outpatient clinic. After measuring height and weight, a full body scan by dual-energy x-ray absorptiometry (DXA) and a BIA measurement was performed. Thirteen predictive FFM-BIA equations based on weight, height, age, resistance, reactance and/or impedance were systematically selected and compared to FFM-DXA. Accuracy of FFM-BIA equations was evaluated by the percentage adolescents predicted within 5 % of FFM-DXA measured, the mean percentage difference between predicted and measured values (bias) and the Root Mean Squared prediction Error (RMSE). Multiple linear regression was conducted to develop a new BIA equation.

**Results:**

Validation was based on 103 adolescents (60 % girls), age 14.5 (sd1.7) years, weight 94.1 (sd15.6) kg and FFM-DXA of 56.1 (sd9.8) kg. The percentage accurate estimations varied between equations from 0 to 68 %; bias ranged from −29.3 to +36.3 % and RMSE ranged from 2.8 to 12.4 kg. An alternative prediction equation was developed: FFM = 0.527 * H(cm)^2^/Imp + 0.306 * weight - 1.862 (R^2^ = 0.92, SEE = 2.85 kg). Percentage accurate prediction was 76 %.

**Conclusions:**

Compared to DXA, the Gray equation underestimated the FFM with 0.4 kg (55.7 ± 8.3), had an RMSE of 3.2 kg, 63 % accurate prediction and the smallest bias of (−0.1 %). When split by sex, the Gray equation had the narrowest range in accurate predictions, bias, and RMSE. For the assessment of FFM with BIA, the Gray-FFM equation appears to be the most accurate, but 63 % is still not at an acceptable accuracy level for obese adolescents. The new equation appears to be appropriate but await further validation. DXA measurement remains the method of choice for FFM in obese adolescents.

**Trial registration:**

Netherlands Trial Register (ISRCTN27626398).

## Background

The prevalence of obesity in adolescents is high and increasing [1, 2]. Accurate assessment of fat mass (FM) and fat-free mass (FFM) in obese adolescents is necessary for establishing reachable goals for healthy weight loss and evaluation of treatment. One of the main objectives of obesity management is to reduce FM and to preserve FFM during weight loss. Especially in adolescents FFM changes will occur, and therefore weight change is less informative. Body composition (FFM and FM) can be assessed by several techniques such as underwater weighing, total body potassium, deuterium dilution and dual energy X-ray absorptiometry (DXA). These methods are time-consuming, expensive; need trained operators and are hardly feasible in most dietetic settings [3, 4]. DXA is acknowledged as the standard [5] and most precise [6] method to assess body fat mass, although it can only be used in special settings and requires the use of a very low dose of radiation [7]. Unlike other methods, DXA measures three components of body composition – bone mineral content, fat tissue mass, and lean tissue mass – as well as regional fat distribution.

In contrast to DXA, bioelectrical impedance analysis (BIA) is a commonly used, safe and simple, portable, non-invasive, inexpensive technique that needs minimal operator training, making it appropriate for use in daily clinical practice. The BIA method is based on the conduction of electrical current in the body and differences in electrical conductivity between the fat and water components of the body. The electrical resistance and reactance together with body weight and height can reliably estimate body composition. But, the results of the BIA is highly dependent on which FFM-BIA equation is used. In order to assess FFM with BIA, several FFM-BIA equations have been developed. Only a few FFM equations have been developed for obese adolescents [8–10]. To the best of our knowledge no studies exist on validation of all available FFM equations in obese Caucasian adolescents. Because of their mean weight and BMI our study group was almost comparable with adults. This is the reason to include also BIA equations based on healthy and obese adults. As part of evidence-based practice, the aim of this study was to 1) examine the validity of published BIA-FFM equations, based on healthy and/or obese population (children and adults), for obese 11–18 year old adolescents using DXA as the reference method and 2) to develop a new FFM-BIA equation for obese adolescents.

## Methods

### Subjects

Adolescents were referred by their general practitioner or school doctor to the outpatient pediatric obesity clinic of the VU University Medical Center Amsterdam. At their first visit the paediatric-endocrinologist interviewed all adolescents concerning their medical history, weight development and ethnicity [11, 12]. The physical examination included height, weight, waist circumference, blood pressure and pubertal Tanner stage [13].

Subjects were eligible when they met the following inclusion criteria: 1) age of 11–18 years; 2) obesity according to the definition of Cole et al. [14]. Cole et al. provide international cut off points for body mass index for overweight and obesity by sex in childhood (2–18 years), based on international data and linked to the widely accepted adult cut off points of a body mass index of 25 and 30 kg/m^2^. Exclusion criteria were: not Dutch-speaking, obesity as a result of a known syndrome or organic cause (hypothyroidism), developmentally delayed , physical limitations (e.g. wheelchair dependent) and diagnosed type 2 diabetes mellitus. Subjects were measured between November 2006 and August 2008.

### Ethics

The medical ethical committee of VU University Medical Center approved the study protocol. Adolescents, as well as their parents, gave written informed consent.

### Anthropometrics

All measurements were performed using the same protocol. After an overnight fast, the subjects attended the outpatient clinic. Height was measured with an accuracy of 0.1 cm with an electronic stadiometer (KERN250D, De Grood Metaaltechniek, Nijmegen, Netherlands). Body weight (WT) was measured (in underwear) within 0.1 kg with a calibrated electronic flat scale (SECA861, Schinkel, Nieuwegein, Netherlands). Weight and height were used to calculate BMI (weight in kilograms divided by the square of height in meters). For the body mass index standard deviation score (BMIsds) reference data of Dutch children collected in 1997, were used (www.growthanalyser.org) and the sds, or z-score, calculated. The BMIsds indicates how many standard deviations a measurement is above or below the mean of the normal distribution.

### Dual energy x-ray absorptiometry

After measuring height and weight, a full body scan was performed by dual-energy x-ray absorptiometry (DXA; Hologic QDR4500-Delphi, software 12.3.3. S/N 45665, Tromp Medical, Castricum). DXA is based on the measurement of the attenuation of a collimated x-ray beam from a source with two energies passing through the body. Subjects (in the fasting state) were scanned for 10 min in underwear while lying in the supine position. The DXA method assessed, total body weight (WTdxa), fat mass (FM) and fat-free mass (FFM) defined as lean tissue mass including bone mineral content.

### Bioelectrical impedance analyzes (BIA)

On the same morning as the DXA and also in the fasting state a BIA measurement was performed. Shoes and socks were removed, and the subjects were in a supine position. The BIA measurements were carried out on the non-dominant side of the subject, using a Hydra ECF/ICF Bio-Impedance Spectrum Analyzer, model 4200 (Xitron Technologies, San Diego, CA, USA). Four electrodes were placed on the hand and foot. For the wrist, one electrode was placed to bisect the ulnar hand, and the other electrode was placed just behind the middle finger. One of the ankle electrodes was placed to bisect the medial malleolus and the other was placed just behind the middle toe. The resistance and reactance measured at 50 kHz were used in the evaluation of BIA-FFM equations, obtained by the program Hydra Data Acquisition Utility.

### BIA-FFM equations

PubMed was systematically searched (through November 2014) for publications on Mesh-derived keywords; *Electric Impedance, Absorptiometry, Photon, body composition, equations and prediction* in every possible combination. Applied limitations were ‘English language’, ‘humans’, not ‘critical illness’, and ‘intensive care’. More references were obtained by screening reference lists of relevant publications. Equations were included when based on impedance or resistance data from BIA, and when the study was performed in a healthy or obese population mean age > 11 years including both males and females. Exclusion criteria were: patients, insufficient information on body assessment method (e.g. FFM based on assumptions), only a specific ethnic group (other than Caucasian), small sample size (*n* < 50), only based on elderly (>60 years), unusual variable in the fat free mass equation (e.g. skinfold, body density, deuterium dilution) and athletes.

### Statistics

Subject characteristics (boys versus girls) were analyzed by independent samples T-test. For each participant, the FFM was predicted by the equations (FFM-BIA) and determined by DXA (FFM-DXA). The percentage of subjects with BIA-FFM predicted within ± 5 % of FFM-DXA was considered as a measure of accuracy at the individual level. This limit was chosen as being consistent with technical measurement errors of 5 % or less [9]. A predicted BIA-FFM below 95 % of FFM-DXA was classified as underestimation and a prediction above 105 % of FFM-DXA was classified as overestimation. The mean percentage difference between the predicted FFM-BIA and FFM-DXA was considered a measure of accuracy on a group level (bias). Also, the maximum values found for negative error (underestimation) and positive error (overestimation) were determined. The root mean squared prediction error (RMSE) was used to indicate how well the equation predicted in our dataset. The RMSE is calculated based on the difference between the BIA predicted value and the DXA reference value, all individual differences squared, taken the mean of the squared differences, and subsequently the root of the mean value [15]. The most accurate equation was defined as follows: the highest level of accurate predictions, with the smallest difference between boys and girls (to find the best fitting equation for both sexes), with the smallest bias, and the smallest RMSE. For the development of a new BIA equation the DEXA-derived FFM was applied as the criterion for the development of a new prediction through multiple regression analysis. The following potentially influencing variables were used: body weight, age, body height, BMI, RI, ZI, R, Z, X, sex (coded as female = 0 and male = 1), and Tanner’s stages [16]. Additionally we have conducted an evaluation of accuracy of the best performing BIA approach as concluded from the baseline evaluation. For 73 subjects (25 boys, 48 girls) we had matching six months follow-up BIA and DXA measurements as part of a weight loss trial [17]. In this way, it was possible to evaluate the values for monitoring the subjects in time. Data were analyzed using SPSS 20.0 and RMSE with Excel.

## Results

A total of 125 adolescents participated in this study. 22 adolescents were excluded because of overweight (*n* = 10), due to a body weight higher than allowed for the DXA (>125 kg) (*n* = 4) or missing data because of defective equipment (BIA) (*n* = 8). Table 1 shows subject characteristics of the 103 (61 girls, 42 boys) adolescents by sex.

**Table 1:** Subject characteristics

	Total (*n* = 103)	Girls (*n* = 61)	Boys (*n* = 42)
	Mean (sd)	Mean (sd)	Mean (sd)
Age, y	14.4 (1.7)	14.7 (1.7)	14.1 (1.7)
Height, m	1.66 (0.09)	1.64 (0.06)	1.69 (0.12)
Weight, kg	94.3 (15.7)	94.3 (14.0)	94.2 (18.0)
Waist circumference, cm	109.0 (11.1)	108.4 (11.1)	109.7 (11.2)
BMI (kg/m^2^)	33.9 (4.1)	34.8 (4.2)	32.7 (3.5)
BMI SDS	2.98 (0.34)	2.94 (0.35)	3.05 (0.32)
Tanner stage, n^b^			
Pre-pubertal (stage 1)	32	10	22
Pubertal (stage ≥ 2)	65	50	15
BIA			
-Resistance, Ω	505.2 (54.1)	508.8 (49.2)	500.0 (60.7)
-Reactance, Ω	62.6 (7.7)	63.9 (7.7)	60.7 (7.5)
-Impedance, (Z)	509.1 (54.3)	512.8 (49.6)	503.7 (60.8)
-Fat-free mass, kg^a^	57.8 (10.1)	55.9 (7.4)	60.4 (12.7)
DXA			
-Fat-free mass, kg	56.1 (9.8)	54.7 (7.1)	58.1 (12.5)

^a^Fat-free mass is supplied by Hydra ECF/ICF Bio-Impedance Spectrum Analyzer

^b^For Tanner stage missing values were 1 for girls and 5 for boys

A total of 55 studies were retrieved examining BIA-FFM equations. Our first search provided 24 citations. Another 31 citations were obtained by screening reference lists of relevant publications. Forty-two papers were excluded due to the mean age <11 year (*n* = 16); one gender (*n* = 4); patients (*n* = 6), insufficient information (*n* = 6); specific ethnic group (*n* = 2); small sample size (*n* < 50) (*n* = 1); only based on elderly (>60 y) (*n* = 1); unusual variable (e.g. body density, deuterium dilution) (*n* = 3); non-standard (standard =50kH) method (1 MHz) or no hand to foot measurement (*n* = 3). Of the thirteen included studies (see Table 2) we selected the best equation per study based on explained variance in regression analysis. Five equations were based on only children <19 y [8, 9, 18–20], only two equations were based on adolescents in the age range of 10–18 years [9, 19]. One equation was based on obese adolescents only [9] and two studies were based on Dutch adolescents [18, 21].

**Table 2:** Predictive equations for fat-free mass based on children and adolescents and/or adults with normal weight, both normal weight and obese and obese only

*Author*; reference (ref), BIA system	N (Male), age range y (n)	Age (y)	Height (cm)	Weight (kg)	FFM predictive equations	Statistics and cross-validation
		Mean (sd)	Mean (sd)	Mean (sd)		
Equations based on healthy non-obese children, adolescents and/or adults						
Deurenberg’91 [18] ref: underwater weighing; BIA-101 (RJL)	827 (361 M), 7−15y (*n* = 166)	28 (17)	169.6 (14.3)	64.8 (17.2)	≤15y: 0.406*10^4^*(H(m)^2^/Imp) + 0.360 W + 5.58H(m) + 0.56SEX – 6.48	R^2^ = 0.97, SEE = 1.68
	16-83y (*n* = 661)				≥16y: 0.340 * 10^4^ * (H(m)^2^/Imp) + 15.34H(m) + 0.273 W – 0.127AGE + 4.56SEX – 12.44	R^2^ = 0.93, SEE = 2.63
Deurenberg’90 [21] ref: underwater weighing; BIA101 (RJL)	246 (130 M); 10-15y: 71 M	12.8 (1.5)	159.0 (1.2)	47.2 (11.8)	0.438*10^4^*(H(m)^2^/R) + 0.308 W + 1.6SEX + 7.04H(m) – 8.5	r = 0.99, SEE = 2.39
	20 F	10.7 (1.0)	144.1 (7.7)	35.0 (6.5)		
	16-25y: 41 M	21.6 (2.8)	183.2 (6.3)	73.1 (5.9)		
	75 F	17.6 (3.6)	168.3 (6.7)	57.9 (9.5)		
Houtkooper [19] ref: deuterium dilution; BIA101 (RJL)	95 (53 M),10-14y	12.3 (1.2)	153.6 (10.6)	47.0 (11.3)	0.61(H^2^/R) + 0.25 W + 1.31	R^2^ = 0.95, SEE = 2.1
Kyle [23] ref: DXA; BIA: Xitron4000b	343 (202 M); 22-94y, 20-29y; 21 M		178.7 (6.8)	75.2 (9.8)	−4.104 + 0.518(H^2^/R) + 0.231 W + 0.130Reac + 4.229SEX	r = 0.986, SEE = 1.72
	22 F		165.4 (4.0)	61.7 (6.0)		
	30-39y; 77 M		178.2 (7.1)	79.1 (10.6)		
	29 F		166.4 (6.0)	61.8 (6.4)		
	40-49y 36 M		177.3 (7.3)	81.5 (8.1)		
	13 F		164.0 (6.7)	63.1 (9.9)		
	50-59y; 15 M		176.1 (4.9)	82.4 (10.5)		
	11 F		163.7 (5.3)	67.1 (11.7)		
	60-69y; 11 M		173.4 (4.5)	77.3 (10.1)		
	18 F		161.9 (6.6)	65.0 (11.2)		
	70-79y; 30 M		174.0 (6.5)	75.5 (9.6)		
	22 F		160.5 (6.2)	65.1 (11.6)		
	>80; 12 M		168.3 (6.1)	72.7 (8.7)		
	33 F		156.5 (3.9)	59.9 (8.6)		
Suprasongsin [33] ref: Isotope dilution (H218O); BIA (RJL)	56 (28 M); age 8-26y				0.524(H^2^/R) + 0.415 W – 0.32	R^2^ = 0.96, SEE = 2.8
	18 prepubertal	10.6 (0.3)	142.1 (2.3)	39.6 (2.7)		
	16 pubertal	13.7 (0.3)	164.7 (2.0)	54.2 (2.1)		
	8 adults	22.0 (0.7)	170.4 (3.1)	67.3 (3.6)		
Equations based on healthy non-obese and obese children, adolescents and adults						
Gray [22] ref: underwater weighing; BIA (RJL)	87 (25 M); 19−74y, M	41	178 (164–198)	99.6 (57.8-179.1)	M: 0.00139H^2^ - 0.0801R + 0.187 W + 39.830	R^2^ = 0.97
	F	41	164 (152–177)	89 (51.0-148.6)	F: 0.00151H^2^ – 0.0344R + 0.140 W - 0.158AGE + 20.387	R^2^ = 0.92
Lukaski [34] ref: underwater weighing; BIA four terminal impedance plethysmograph (RJL)	114 (47 M), 18-50y, M	26.9 (8.0)	182.4 (9.1)	86.0 (16.4)	0.756(H^2^/R) + 0.110 W + 0.107Reac - 5.463	r = 0.99, SEE = 2.3
	F	27.0 (6.4)	166.3 (8.3)	61.8 (10.4)		
Scheafer [20] ref: 40 K spectrometry, BIA (Holtain Ltd)	112 (59 M), 3.9-19.3y,	11.8 (3.7)	150.2 (19.7)	42.8 (16.6)	0.65(H^2^/Imp) + 0.68AGE + 0.15	R^2^ = 0.975, RMSE = 1.98
Sun [35] ref :4-C model hydrostatic weighing, deuterium dilution and DXA; BIA RJL	1304; 12-94y, 412 white M	41.9 (20.1)	174.3 (11.1)	75.6 (16.2)	M:-10.68 + 0.65(H^2^/R) + 0.26 W + 0.02R	R^2^ = 0.90, RMSE = 3.9
	114 black M	48.3 (19.3)	173.7 (8.6)	79.9 (15.4)		
	622 white F	42.4 (19.5)	162.9 (8.1)	65.4 (15.6)	F: −9.53 + 0.69(H^2^/R) + 0.17 W + 0.02R	R =0.83, SEE 2.9
	156 black F	51.7 (18.4)	161.1 (8.7)	73.5 (17.1)		
Equations based on healthy obese children, adolescents and adults						
Haroun [10] ref: 3 C model (deuterium, BODPOD and WT; BIA: Tanita BC-418 MA)	78 (30 M), 5-22y				−2.211 + 1.115(H^2^/Imp)	R^2^ = 0.96, SEE = 2.31
	M	12.0 (3.4)	152.4 (16.7)	65.1 (20.6)		
	F	11.3 (3.5)	148.0 (13.7)	60.1 (16.3)		
Horie [36] ref: ADP (BODPOD) and FourF-BIA (Quadscan)	119 (36 M); M, age 18-62y, F (preoperative gastric bypass patients)	38.5 (11.7)	152.8 (25.1)	174.8 (8.2)	WT – (23.25 + 0.13AGE + 1 W + 0.09R – 0.80H)	R^2^ = 0.973
		42.9 (11.4)	114.9 (17.6)	158.7 (6.9)		
Lazzer [9] ref: DXA; BIS (Human IM plus II)	58 (27 M), 10-17y, severely obese subjects	14.2 (1.9)	164.0 (10.0)	92.5 (14.5)	0.87(H^2^/Imp) + 3.1	r = 0.91, RMSE = 2.7
Wabitsch [8] ref: Deuterium dilution (labeled water); BIA RJL	146 (68 M), 5-17y; obese white subjects	12.7 (3.0)	158.5 (15.7)	74.1 (22.3)	(0.35(H^2^/R) + 0.27AGE + 0.14 W – 0.12)/0.732	R^2^ = 0.96, SEE = 1.91

*M* male; *F* female; *T* total (man and female); *BIA* bioelectrical impedance analysis; *DXA* dual-energy x-ray absorptiometry; *W* weight in kg; *H* height in cm; *H(m)* height in meters; *AGE* age in y ; *R* Resistance; *Reac* Reactance; *Imp* Impedance; *FFM* fat-free mass; SEX (M = 1,F = 0)

The FFM average for the entire study group measured by DXA was 56.1 sd 9.8 kg. Table 3 provides the FFM data as mean measured FFM in kg, the percentage of accurate under- and overestimation, the percentage bias, the maximum values found for negative error (underestimation) and positive error (overestimation) and the RMSE in kg. The percentage accurate estimations varied between equations from 0 to 68 %. The bias for equations varied from −21.5 % to +21.6 % and RMSE varied from 2.9 to 13.5 kg. Individual errors were much higher as shown by maximum negative and maximum positive error.

**Table 3:** Evaluation of Fat-Free Mass predictive equations in 103 Dutch obese adolescents, based on bias, RMSE, and percentage accurate prediction, sorted by % accurate prediction

REE predictive equation	FFM^a^	SD	Accurate predictions^b^	Under predictions^c^	Over predictions^d^	Bias^e^	Maximum negative error^f^	Maximum positive error^g^	RMSE^h^
	Kg/d		%	%	%	%	%	%	kg
FFM-DXA	56.1	9.8							
Deurenberg’90 [21]	57.1	9.2	68.0	4.9	27.2	2.2	−14.0	12.6	2.8
Gray [22]	55.7	8.3	63.1	18.4	18.4	−0.1	−16.4	12.0	3.2
Kyle [23]	56.4	8.4	61.2	15.5	23.3	1.2	−10.3	17.2	3.1
Lukaski [34]	53.7	8.5	54.4	39.8	5.8	−3.8	−17.3	11.0	3.9
Sun [35]	57.1	9.6	49.5	16.5	34.0	2.2	−11.2	18.8	3.8
Houtkooper [19]	58.9	9.4	44.7	2.9	52.4	5.4	−12.8	19.2	4.0
Deurenberg’91 [18]	58.8	9.3	40.8	2.9	56.3	5.3	−10.7	17.8	3.9
Haroun [10]	59.5	10.9	35.9	7.8	56.3	6.2	−13.0	29.7	5.7
Lazzer [9]	51.2	8.5	28.2	69.9	1.9	−8.3	−23.2	9.0	6.4
Horie	62.5	9.5	17.5	1.0	81.6	12.0	−5.4	29.7	7.4
Wabitsch [8, 36]	49.8	7.6	7.8	92.2	0.0	−10.7	−25.1	3.0	7.1
Schaefer [20]	45.9	6.8	1.9	98.1	0.0	−17.6	−29.3	−0.7	11.0
Suprasongsin [33]	68.0	11.0	1.0	0.0	99.0	21.6	0.1	36.2	12.4

^a^As measured

^b^The percentage of subjects predicted by this predictive equation within 5 % of the measured value

^c^The percentage of subjects predicted by this predictive equation < 5 % of the measured value

^d^The percentage of subjects predicted by this predictive equation > 5 % of the measured value

^e^Mean percentage error between predictive equation and the measured value

^f^The largest underprediction that was found with this predictive equation as a percentage of the measured value

^g^The largest over prediction that was found with this predictive equation as a percentage of the measured value

^h^Root mean squared prediction error

**Fig. 1:**
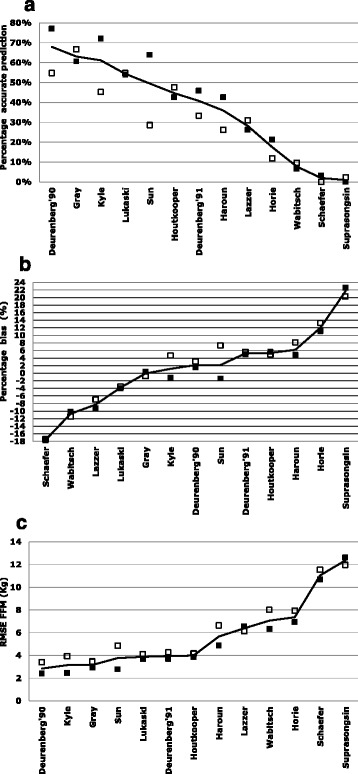
Percentage of accurate predictions (**a**), percentage bias (**b**), and root mean squared prediction error (RMSE) (**c**) for 18 fat-free mass predictive equations in obese girls ■ (*n* = 61) and boys □ (*n* = 42). For each panel, the data are sorted by mean values of all adolescents (line)

Figure 1 shows the percentage of accurate predictions (based on an FFM-BIA within ± 5 % of FFM-DXA), percentage bias, and RMSE for the total group of adolescents by sex. For the total group of adolescents the Deurenberg’90 equation had the smallest RMSE (2.9 kg), 68 % accurate predictions (with 4 % underprediction and 28 % over-prediction) and a bias of 2.5 %. The Gray equation had an RMSE of 3.2 kg, 63 % accurate prediction (with 18 % underprediction and 18 % over prediction) and the smallest bias (−0.1 %). The Kyle equation had an RMSE of 3.1 kg, 61 % accurate prediction (16 % underprediction, 23 % over prediction and a bias of 1.2 %). When split by sex, the Gray equation had the narrowest range in accurate predictions, bias, and RMSE.

Subjects were adolescents and therefore FFM measured at six months after baseline had increased by 1.49 + 2.72 kg as observed by DXA. The best performing BIA equation (the Gray equation) underestimated the FFM change with (0.98 + 2.90 kg; *p* = 0.037).

### Development of new FFM-BIA equation for obese adolescents

H(cm)^2^/Imp was identified as the strongest predictor of FFM (R^2^ = 0.82; *P* < 0.0001). When both H(cm)^2^/Imp and bodyweight were included in the regression model, the explained variance (R^2^) was 92 %. Other variables did not further improve explained variance, which was consistently high for subgroups: sex (R^2^ girls 0.89, boys 0.93), puberty (R^2^ early 0.92, late 0.89). The new FFM-BIA equation was: FFM = 0.527 * H(cm)^2^/Imp +0.306*weight −1.862 (R2 = 0.92, SEE = 2.85). Accurate predictions within this study group was 76 %, however this equation awaits external validation.

## Discussion

To our knowledge this is the first study evaluating all relevant available BIA-FFM equations for assessment of FFM in obese adolescents. We ranked BIA-FFM equations in the percentage of obese adolescents whose FFM was assessed within a reasonable range of error. The equations proposed by Deurenberg’90 et al. [21], Gray et al. [22], and Kyle et al. [23] were able to assess FFM in 61-68 % of the obese adolescents within 5 % of the DXA assessment. This study shows that for the assessment of FFM in obese adolescents, DXA and the BIA-FFM equations are not interchangeable. Some BIA-FFM equations perform better than others, but all lack accuracy. The Gray equations performed best. Our new equation predicted FFM in 76 % of the obese adolescents in our study group. However, the equation awaits external validation. There is no consensus regarding the level of error that is acceptable when measuring FFM. In theory the 2.5 % cut-off value is clinically more appropriate than the 5 % cut-off value, since an error of about 3 kg (see Table 2: 5 % of FFM-DXA (56,1 kg) = 3 kg) appears quite large. In case a cut-off of 2.5 % was used, the maximum accuracy level found was 35 %. However, in this study the cut-off level was mainly used to rank the available FFM-BIA equations from good to poor.

There is a whole range of published FFM-BIA equations, although only three originally developed in obese adolescents [8–10]. However, all three failed to produce acceptable FFM values comparable to DXA when applied to our sample of obese adolescents. Therefore, in this study, other FFM-BIA equations were considered, both based on a larger range of children with respect to age as well as weight and BMIsds. In an earlier study on energy expenditure we showed that obese adolescents have such stature and body mass that equations relevant for energy expenditure should rather be based on the 18+ category and not the normal 12–18 year age range [24]. In fact, the Gray equation is developed in adults (25 m, 62f), with BMI varying from 19.6 tot 53.3 kg/m2 and percent body fat from 8.8 tot 59 %. Compared to DXA in our study, the BIA algorithm that is part of the devices overestimated FFM in obese children and adolescents [9, 25]. In healthy adult persons, the assumption is that 73.2 % of the lean body mass consists of total body water [26]. Wells et al. found that in children (aged 4–23 y) the FFM hydration is higher (mean 75 %), and differed by age and sex [27]. In obese, the hydration of the lean body mass is also great er than 73.2 %. This increase in hydration in obese compared with non-obese individuals averaged 1 % and reached 2 % in extreme obesity [28]. So both, childhood and obesity could cause an overestimation of FFM, which in turn could underestimate FM. As far as we known, all selected developed FFM equations (see Table 2) are based on the assumption of the hydration factor of 73.2 %. So far, it is unclear whether FFM equations based on adults could be adapted for use in children by correction factor for hydration.

DXA is still the gold standard to measure body composition [29]. The current evaluation, in line with others [25, 30], neglects measurement error by DXA methodology [31] and ascribes all error, being the deviation between BIA and DXA, to the BIA methodology. Measurement error in FM (%) can also result from inaccurate detection of FM in the trunk region or variation in tissue thickness [28]. Alternatively, an individual subject may have an overestimation of FFM by DXA and at the same time an underestimation by BIA-FFM [32]. Strengths of this study include the use of DXA, a robust and well-accepted measure [29] and the systematic literature search of FFM-BIA equations.

A limitation of the study is that the interpretation is not applicable to other BIA and DXA devices or software. Besides this our DXA had a limit of 125 kg. Larger individuals can currently be measured depending on the weight capacity and scanning area of the instrumentation. It is unknown whether our results can be extrapolated to the most obese adolescents. The new equation awaits external validation, as this was not possible in the present study due to the small sample.

## Conclusions

In conclusion, the present study shows that DXA and BIA-FFM equations are not interchangeable for the assessment of FFM in obese adolescents. There is a wide variation in the accuracy of predictive equations for FFM in obese adolescents. Compared to DXA, FFM of two out of three adolescents was accurately predicted using the Gray equation based on weight, height, age, sex, and resistance index.

### Ethical approval

This study was approved by the ethical committee for human studies of the VU University Medical Center Amsterdam. The adolescents, as well as their parents, gave written informed consent.

## Abbreviations

BIA: Bioelectrical impedance analysis
DXA: Dual energy x-ray absorptiometry
FFM: Fat free mass
FM: Fat mass
FFM-BIA: FFM calculated by BIA equation
FFM-DXA: FFM measured by dual energy x-ray absorptiometry
BMIsds: Body Mass Index standard deviation score
RMSE: Root Mean Squared prediction error
